# Gender Diversity and Brain Morphology Among Adolescents

**DOI:** 10.1001/jamanetworkopen.2023.13139

**Published:** 2023-05-12

**Authors:** Yllza Xerxa, Tonya White, Samantha Busa, Leonardo Trasande, Manon H. J. Hillegers, Vincent W. Jaddoe, Francisco Xavier Castellanos, Akhgar Ghassabian

**Affiliations:** 1 Department of Child and Adolescent Psychiatry, Erasmus University Medical Center, Rotterdam, the Netherlands; 2 Department of Radiology and Nuclear Medicine, Erasmus University Medical Center, Rotterdam, the Netherlands; 3 Generation R Study Group, Erasmus University Medical Center, Rotterdam, the Netherlands; 4 Section on Social and Cognitive Developmental Neuroscience, National Institute of Mental Health, Bethesda, Maryland; 5 Department of Child and Adolescent Psychiatry, New York University Grossman School of Medicine, New York, New York; 6 Department of Pediatrics, New York University Grossman School of Medicine, New York, New York; 7 Department of Population Health, New York University Grossman School of Medicine, New York, New York; 8 Department of Environmental Medicine, New York University Grossman School of Medicine, New York, New York; 9 Department of Epidemiology, Erasmus University Medical Center, Rotterdam, the Netherlands; 10 Department of Pediatrics, Sophia Children’s Hospital, Erasmus University Medical Center, Rotterdam, the Netherlands

## Abstract

**Importance:**

Gender-diverse youths have higher rates of mental health problems compared with the general population, as shown in both clinical and nonclinical populations. Brain correlates of gender diversity, however, have been reported only among youths with gender dysphoria or in transgender individuals.

**Objective:**

To examine brain morphologic correlates of gender diversity among adolescents from a general pediatric population who were assigned male or female at birth, separately.

**Design, Setting, and Participants:**

This cross-sectional study was embedded in Generation R, a multiethnic population-based study conducted in Rotterdam, the Netherlands. Adolescents who were born between April 1, 2002, and January 31, 2006, and had information on self-reported or parent-reported gender diversity and structural neuroimaging at ages 13 to 15 years were included. Data analysis was performed from April 1 to July 31, 2022.

**Exposures:**

Gender-diverse experiences among adolescents were measured with selected items from the Achenbach System of Empirically Based Assessment forms and the Gender Identity/Gender Dysphoria Questionnaire for Adolescents and Adults, as reported by adolescents and/or their parents.

**Main Outcomes and Measures:**

High-resolution structural neuroimaging data were collected using a 3-T magnetic resonance imaging scanner (at a single site). We used linear regression models to examine differences in global brain volumetric measures between adolescents who reported gender diversity and those who did not.

**Results:**

This study included 2165 participants, with a mean (SD) age of 13.8 (0.6) years at scanning. A total of 1159 participants (53.5%) were assigned female at birth and 1006 (46.5%) were assigned male at birth. With regard to maternal country of origin, 1217 mothers (57.6%) were from the Netherlands and 896 (42.4%) were from outside the Netherlands. Adolescents who reported gender diversity did not differ in global brain volumetric measures from adolescents who did not report gender diversity. In whole-brain, vertexwise analyses among adolescents assigned male at birth, thicker cortices in the left inferior temporal gyrus were observed among youths who reported gender diversity compared with those who did not. No associations were observed between gender diversity and surface area in vertexwise analyses.

**Conclusions and Relevance:**

The findings of this cross-sectional study suggest that global brain volumetric measures did not differ between adolescents who reported gender diversity and those who did not. However, these findings further suggest that gender diversity in the general population correlates with specific brain morphologic features in the inferior temporal gyrus among youths who are assigned male at birth. Replication of these findings is necessary to elucidate the potential neurobiological basis of gender diversity in the general population. Future longitudinal studies should also investigate the directionality of these associations.

## Key Points

**Question:**

Is gender diversity associated with specific brain morphologic features among adolescents in the general population?

**Findings:**

In this cross-sectional study of 2165 adolescents from the Netherlands general population, no significant differences in total brain volumetric measures were observed between youths who reported gender diversity and youths who did not.

**Meaning:**

These findings shed light on the role of biology in the development of gender diversity and may contribute to destigmatization, greater acceptance, and improved quality of life for individuals with diverse gender identities.

## Teaser

This cross-sectional study examines brain morphology correlates of gender diversity among adolescents from a general pediatric population in the Netherlands.

## Introduction

Adolescence marks an important developmental period and a transition point for girls and boys, especially with regard to consolidating gender identity.^1,2,3^ Through this process, an increasing number of adolescents report gender diversity, defined as the extent to which a person’s gender identity, role, or expression differs from the cultural norms prescribed for people of a particular sex.^4^ Gender-diverse youths have higher rates of mental health problems compared with the general population, as shown in both clinical and nonclinical populations.^5,6,7,8,9^ While it is not fully clear how gender diversity places adolescents at greater risk for mental health problems, minority stress theory suggests that stigmatization, discrimination, and bullying serve as key underlying risk factors for the high degree of stress. In addition, prenatal influences on brain development during the fetal period may contribute to both mental health outcomes and gender diversity.^10,11^

Our knowledge of the neurobiology of gender diversity is mainly based on a few studies of transgender adults with inconclusive findings. Magnetic resonance imaging (MRI) studies of transgender individuals (particularly transgender women) have reported larger putamen volumes compared with individuals with matching gender identity and assigned sex at birth.^12,13^ Another study did not find differences in cortical thickness between transgender women and cisgender women or men.^14^ One study in adolescents reported that in the cerebellum and hypothalamus, transgender youths were more similar to those who shared their gender identity.^15^ Data from the ENIGMA Transgender Persons Working Group showed that transgender individuals differed from individuals who had matching gender identity and assigned sex at birth with respect to subcortical brain volumes and surface area, but not cortical thickness.^16^ The ENIGMA study, however, did not include children and adolescents. Furthermore, about half of adolescents who report gender diversity do not identify as transgender.^17^ Thus, additional opportunities for developmental research with gender-diverse populations include studies of gender-diverse adolescents from the general population who may or may not identify as transgender.

Considering the many different ways in which gender diversity can shape human experience and also its potential clinical implications,^7,8^ understanding the underlying neurobiology can shed further light on elevated risks for mental health problems in gender-diverse adolescents. Boys and girls differ in both brain structure and function, and these differences increase during development.^18^ Prior research suggests that organizational effects of sex steroids on the brain during later stages of fetal life may underlie gender development.^19,20^ Whether gender diversity in the general population is associated with specific brain morphology or function is yet to be investigated.

Here, we examined brain morphologic correlates of gender diversity in adolescents from the general population in the Netherlands. Since studies in transgender individuals have reported different findings in transgender women and men, we a priori examined brain correlates of gender diversity in adolescents assigned male or female at birth, separately. We examined global brain volumetric measures (cerebrum and cerebellum), structures implicated in the literature (thalamus, amygdala, and hippocampus), and vertexwise cortical thickness. In this study, we relied on 2 items (“wished to be the opposite sex” and “would rather be treated as someone from the opposite sex”) to ascertain gender diversity.

## Methods

### Participants

This cross-sectional study was approved by the Medical Ethics Committee of the Erasmus Medical Center. Written informed consent was obtained from all adult participants and from both parents of minors. Participants gave written informed consent for each phase of the study (fetal, preschool, childhood, and adolescent periods). Children received oral information about the study and provided assent after age 12 years. This study followed the Strengthening the Reporting of Observational Studies in Epidemiology (STROBE) reporting guideline.

Our cross-sectional research was embedded in Generation R, a multiethnic population-based cohort study from fetal life onward.^21^ Briefly, pregnant women living in Rotterdam, the Netherlands, with an expected delivery date between April 1, 2002, and January 31, 2006, were invited to participate. Children and their families were assessed repeatedly using surveys and in-person measurements, including repeated brain and body MRI assessments.^22^

At ages 13 to 15 years, 4512 adolescents and/or their parents reported on gender diversity. Our measure of gender diversity did not adequately cover the full spectrum, as we relied only on 2 items and did not directly measure gender identity or expression using questionnaires. In this group, neuroimaging assessment using MRI was available for 3414 adolescents. After we excluded an additional 1126 participants with poor imaging quality, incidental findings, dental braces, and 1 child of 36 twin pairs and 87 sibling pairs, our final sample consisted of 2165 adolescents with data on gender diversity and structural neuroimaging. This data exclusion vs inclusion was not related to any imbalance with regard to gender diversity.

### Gender Diversity

We selected items from the Achenbach System of Empirically Based Assessment (ASEBA) forms and the Gender Identity/Gender Dysphoria Questionnaire for Adolescents and Adults (GIDYQ)^23^ to assess gender diversity among adolescents. From the Child Behavior Checklist and the Youth Self-Report for older children (CBCL/6-18 and YSR/11-18),^24,25^ we used item 110 that stated “wish to be the opposite sex,” with choices 0 (“not true”), 1 (“somewhat or sometimes true”), and 2 (“very true or often true”), based on the preceding 2 months. From the GIDYQ, we selected the question: “Would you rather be treated as someone from the opposite sex?” Adolescents responded “no,” “probably yes,” or “definitely yes.” We created binary responses by combining “somewhat or sometimes true” and “very true or often true” and “probably yes” and “definitely yes” for the ASEBA and GIDYQ, respectively, and defined adolescents as gender diverse if any item was endorsed by parents and/or adolescents.^26^ Alternatively, we created an ordinal variable with responses of “somewhat or sometimes true” and “very true or often true” and “probably yes” and “definitely yes” separately and reran the models. In our study, adolescents were not specifically asked whether they identified as transgender or if they had socially transitioned.

### Image Acquisition and Processing

Neuroimaging data were acquired using an 8-channel, receive-only head coil on a 3-T GE Discovery MR750W MRI system (GE Healthcare).^22^ Adolescents could participate in a mock scanning session before scanning to become familiar with the procedure and scanning environment. After a 3-plane localizer scan, structural MRI was acquired using a high-resolution T1-weighted coronal inversion recovery–fast spoiled gradient recalled sequence (GE option BRAVO; repetition time, 8.77 ms; time to echo, 3.4 ms; inversion time, 600 ms; flip angle, 10°; matrix size, 220 × 220; field of view, 220 × 220 mm; slice thickness, 1 mm; and autocalibrating reconstruction for Cartesian imaging acceleration factor, 2).^27^

Structural MRI data were processed with the FreeSurfer analysis suite, version 6.0 (FreeSurfer).^28^ After converting the DICOM (Digital Imaging and Communications in Medicine) data to the MGZ file format using the FreeSurfer mri_convert tool, cortical reconstruction and volumetric segmentation was conducted. Specifically, nonbrain tissue was removed, voxel intensities were normalized for the B1 field in homogeneities, and voxels were segmented into gray matter, white matter, and cerebral spinal fluid. Automatic subcortical segmentation was also performed, and volumes in cubic millimeters were extracted for the hippocampus and amygdala—the subcortical structures of interest in this study. Our group has developed a metric of image quality that automatically characterizes the amount of motion and/or artifact based on signal intensities outside of the brain.^29^ We additionally controlled for this metric that quantifies motion artifacts and quality. Global metrics of volume were extracted (total brain volume and subcortical volume), and a number of subcortical and cortical structures (eg, amygdala, hippocampus) were automatically labeled.

### Covariates

Date of birth (to calculate a child’s age) and assigned sex at birth were obtained from birth records. Maternal age and paternal age were assessed at enrollment during pregnancy. Mothers were asked about their country of origin at enrollment. Given the diversity of Rotterdam and its surrounding area, participants reported a wide spectrum of ethnic origins, including Cape Verdean, Dutch, Dutch Antillean, Moroccan, Surinamese, Turkish, and several other non-Dutch African, Asian, European, North American, and South American ethnicities. We operationalized these data following prior work in the Generation R Study as a 2-category variable we refer to as “maternal national origin.” The categories include Dutch and non-Dutch (European [non-Turkish], Moroccan, Surinamese, Turkish, and other ethnicity or national origin). Maternal education was classified in 3 levels: low (maximum of 3 years of general secondary school), medium (intermediate vocational training or >3 years of general secondary school), and high (bachelor degree or higher academic education). In surveys of individuals aged 13 to 15 years, self-perceived puberty was assessed with the puberty developmental scale,^30^ a validated self-report questionnaire with 5 items answered on a 5-point scale.

### Statistical Analysis

Descriptive statistics are presented for adolescent and parent reports of gender diversity at age 13 to 15 years. We used linear regression models to examine differences in global brain volumetric measures (total brain volume, gray matter volumes, and cerebral white matter volumes) between adolescents who reported gender diversity and those who did not. We further examined differences between the 2 groups in subcortical brain measures stratified by hemispheres with separate linear regressions. Analyses defined gender diversity as the independent variable and adolescent brain measures as dependent variables. Unstandardized regression coefficients (β) and 95% CIs were calculated in all models. Models were adjusted for potential confounding variables, including maternal age, education, and country of origin and child age and puberty developmental scale score. We ran all models stratified by assigned sex at birth. The false discovery rate was applied to adjust for multiple comparisons (6 comparisons, including 3 outcomes among adolescents assigned male or female at birth).^31^ Further, the false coverage rate (FCR) was applied to adjust for multiple comparisons of CIs as follows: FCR = No. of nonconverging CIs/No. of CIs constructed.^32^

Next, surface-based exploratory analyses of cortical thickness and surface area were performed to study the spatial distributions of the associations between gender diversity and brain morphology along the cortex using vertexwise analyses in R, version 4.1.2 (R Project for Statistical Computing), with the QDECR package. Resulting *P*-value maps were corrected for multiple comparisons at the vertex level using gaussian Monte Carlo simulations.^33^ Surface-based analyses of cortical thickness may show nongaussian patterns of spatial correlations, which would increase the false-positive rate greater than .05. We therefore set the cluster-forming threshold to *P* = .001, as this has shown high correspondence with actual permutation testing across all surface measures.^34^ We applied Bonferroni correction to account for analyzing both hemispheres separately (ie, *P* < .025 clusterwise).

Data on maternal education were missing for 166 participants (7.7%), country of origin for 52 (2.4%), and self-reported puberty for 537 (32.9%). We performed multiple imputation that yielded unbiased parameter estimates using chained equations with 20 imputed data sets to account for missing values in the potential confounders.^35^ Statistical analyses were performed using R software, version 4.1.2. Data analysis was performed from April 1 to July 31, 2022.

## Results

This cross-sectional study included 2165 adolescents, with a mean (SD) age of 13.8 (0.6) years at scanning. A total of 1159 participants (53.5%) were assigned female at birth and 1006 (46.5%) were assigned male at birth. Descriptive statistics for participant characteristics are presented in Table 1. With regard to maternal country of origin, 1217 mothers (57.6%) were from the Netherlands and 896 (42.4%) were from outside the Netherlands. Of 1878 adolescents, 82 (4.6%) reported that they either sometimes or very often wished to be the opposite sex. At the same age, 29 of 1582 adolescents (1.8%) reported that they would rather be treated as someone from the opposite sex. When we combined parent and child reports, some experience of gender diversity was endorsed for 96 of 2065 participants (5.1%).^26^ Adolescents who were assigned female at birth were more likely to report gender diversity compared with those assigned male. The mean (SD) age of self-reported puberty during assessment was 13.6 (0.8) years; the mean (SD) age was 13.9 (0.64) years for adolescents assigned male and 13.2 (0.61) years for adolescents assigned female at birth.

**Table 1.** Participant Characteristics^a^

Characteristic	Gender diversity at ages 13-15 y							
	Wish to be opposite sex, parent-report (n = 1924)		Self-report				Overall gender diversity, parent or child report (N = 2165)	
			Wish to be opposite sex (n = 1878)		Rather be treated as opposite sex (n = 1582)			
	No (n = 1904)	Yes (n = 10)	No (n = 1796)	Yes (n = 82)	No (n = 1553)	Yes (n = 29)	No (n = 2069)	Yes (n = 96)
Child age at assessment, mean (SD), y	13.8 (0.6)	13.6 (0.6)	13.6 (0.4)	13.6 (0.4)	13.6 (0.4)	13.6 (0.4)	13.8 (0.6)	13.8 (0.6)
Assigned sex at birth								
Female	1152 (53.6)	7 (85.7)	1105 (53.5)	52 (67.6)	1118 (54.1)	25 (90.9)	1092 (52.9)	67 (69.8)
Male	1003 (46.4)	3 (14.3)	980 (46.5)	28 (32.4)	1018 (45.9)	4 (9.1)	977 (47.1)	29 (30.2)
Child puberty scale, mean (SD)^b^	2.4 (0.7)	2.6 (1.1)	2.4 (0.7)	2.8 (0.7)	2.4 (0.7)	3.0 (0.6)	2.4 (0.8)	2.7 (0.7)
Maternal country of origin								
The Netherlands	1210 (61.1)	7 (71.4)	1167 (61.3)	50 (61.6)	1182 (63.2)	18 (54.5)	1160 (60.7)	57 (60.0)
Outside the Netherlands	893 (38.9)	3 (28.6)	865 (38.7)	29 (38.4)	873 (36.8)	11 (45.5)	858 (39.3)	38 (40.0)
Education level								
High	1021 (53.9)	5 (57.1)	989 (54.3)	37 (49.3)	994 (56.9)	19 (70.0)	978 (53.6)	48 (54.5)
Middle	610 (30.6)	4 (28.6)	586 (30.3)	28 (37.3)	600 (29.7)	6 (20.0)	586 (30.6)	28 (31.8)
Low	478 (15.5)	1 (14.3)	349 (15.4)	10 (13.4)	349 (13.4)	2 (10.0)	347 (15.8)	12 (13.6)
Maternal age at pregnancy, mean (SD), y	31.4 (4.7)	31.1 (4.6)	31.4 (4.6)	31.1 (5.1)	31.1 (4.9)	31.6 (4.2)	31.4 (4.7)	30.1 (5.0)

^a^ Unless indicated otherwise, values are presented as No. (%) of participants. Data on maternal education were missing for 166 participants (7.7%), country of origin for 52 (2.4%), and self-reported puberty for 537 (32.9%).

^b^ Range, 0 to 4, where a higher score indicates more advanced development of puberty.

Adolescents who reported gender diversity did not differ in global brain volumetric measures from adolescents who did not report gender diversity (Table 2). However, among youths who were assigned female, those who reported gender diversity had smaller thalamic volumes in both the left hemisphere (β = −0.16 [95% CI, −0.28 to −0.04]) and right hemisphere (β = −0.14 [95% CI, −0.25 to −0.03]) compared with adolescents who did not report gender diversity (Table 3). These associations did not remain after adjustment for multiple comparisons. Moreover, we observed no difference in comparisons of CIs and the underlying *P* values after adjusting for the FCR. Similarly, among youths who were assigned female, gender-diverse adolescents had smaller right hemisphere cerebellar volumes (β = −0.34 [95% CI, −0.63 to −0.05]) compared with those who did not report gender diversity. This association also did not survive correction for multiple comparisons. We observed no associations with other subcortical brain regions and no association with gender diversity overall in adolescents assigned male at birth (Table 3).

**Table 2.** Brain Morphology and Adolescent Gender Diversity^a^

Adolescents with gender-variant experiences, assigned sex at birth (n = 96)	Global brain volumetric measure, cm^3^ (N = 2165)					
	Total brain volume		Cerebral white matter volume		Total gray volume	
	β (95% CI)^b^	*P* value	β (95% CI)^b^	*P* value	β (95% CI)^b^	*P* value
Female	−3.17 (−10.7 to 6.49)	.15	−3.94 (−10.6 to 9.89)	.32	3.46 (−9.10 to 16.0)	.59
Male	5.75 (−8.44 to 9.18)	.60	4.87 (−12.6 to 22.3)	.58	0.88 (−19.3 to 21.1)	.93

^a^ Models were adjusted for child age at brain scan and child puberty and for maternal age, country of origin, and education.

^b^ Unstandardized regression coefficients (β) refer to the cubic centimeter change in volumetric measures in adolescents who expressed having gender diversity compared with adolescents who did not. The β values were averaged from 20 imputed data sets.

**Table 3.** Associations of Gender Diversity and Subcortical Brain and Cerebellar Volumetric Measures^a^

Adolescents with gender variant experiences, assigned sex at birth (n = 96)	Brain volumetric measure, cm^3^ (N = 2165)										
	Thalamus			Cerebellum			Amygdala			Hippocampus	
	β (95% CI)^b^	*P* value	β (95% CI)^b^		*P* value	β (95% CI)^b^		*P* value	β (95% CI)^b^		*P* value
**Female**											
Left volumetric measures	−0.16 (−0.28 to −0.04)	.01^c^	−0.28 (−0.50 to 0.07)		.62	−0.02 (−0.06 to 0.01)		.22	−0.02 (−0.09 to 0.05)		.51
Right volumetric measures	−0.14 (−0.25 to −0.03)	.01^c^	−0.34 (−0.63 to −0.05)		.02^c^	−0.004 (−0.04 to 0.03)		.85	−0.03 (−0.12 to 0.04)		.41
**Male**											
Left volumetric measures	−0.03 (−0.23 to 0.18)	.80	−0.09 (−0.56 to 0.37)		.71	−0.03 (−0.03 to 0.09)		.46	−0.004 (−0.11 to 0.12)		.94
Right volumetric measures	0.004 (−0.17 to 0.18)	.96	0.12 (−0.35 to 0.59)		.61	0.02 (−0.04 to 0.09)		.53	−0.06 (−0.19 to 0.06)		.29

^a^ Models were adjusted for child age at brain scan and child puberty and for maternal age, country of origin, education, and total intracranial volume.

^b^ Unstandardized regression coefficients (β) refer to the cubic centimeter change in volumetric measures in adolescents who expressed having gender-variant experiences compared with adolescents who did not. The β values were averaged from 20 imputed data sets.

^c^ These associations did not survive multiple comparisons using the false discovery rate.

In whole-brain, vertexwise analyses of cortical thickness, we observed that among youths who were assigned male at birth, gender-diverse adolescents had thicker cortices in the left inferior temporal gyrus than those who did not report gender diversity (Figure). No cluster was found in the right temporal lobe. We observed no associations between gender diversity and cortical thickness among youths assigned female at birth. No associations were observed between gender diversity and surface area in the vertexwise analysis.

**Figure.**
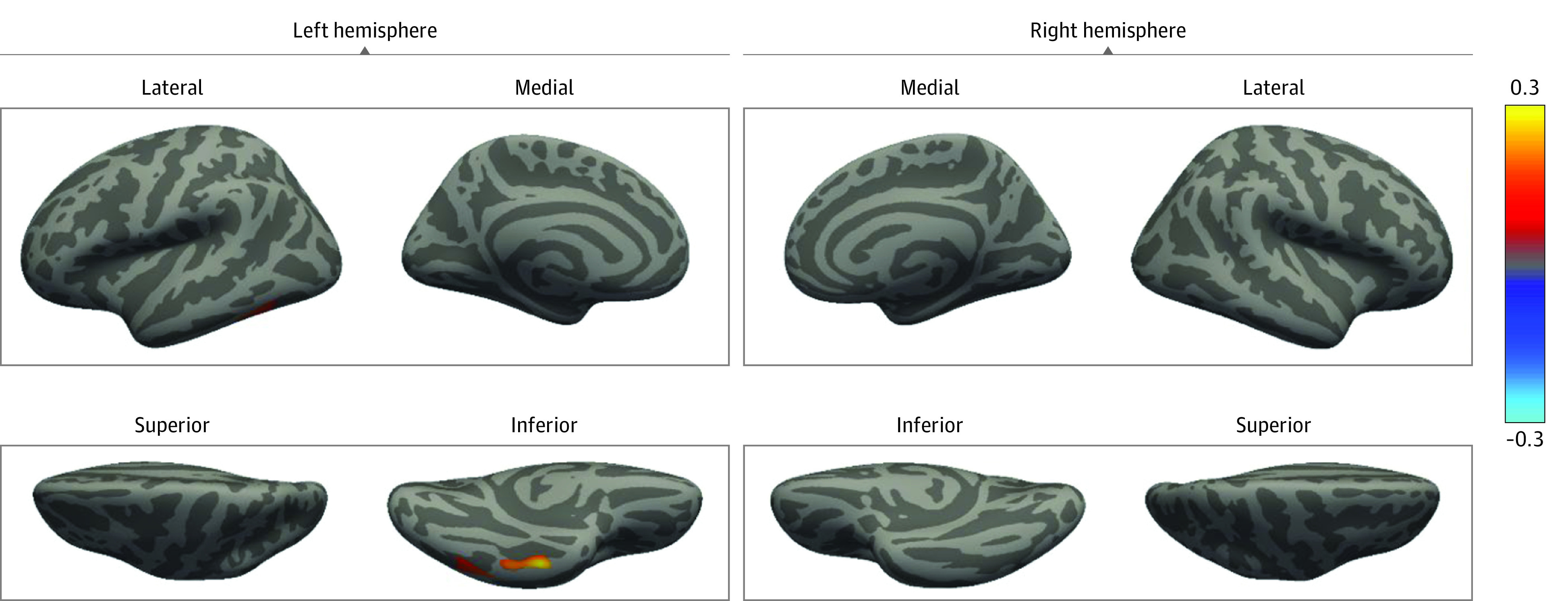
Vertexwise Association Between Gender Diversity and Cortical Thickness Images show clusters from the left and right hemispheres of the brain comparing adolescents assigned as boys at birth who reported gender diversity and who did not, adjusted for child age at brain magnetic resonance imaging, puberty, maternal age, country of origin, and education. The color bar represents a logarithmic scale of *P* values; red and yellow indicate a thicker cortex, and blue and light blue indicate a thinner cortex.

## Discussion

In this cross-sectional study, we examined brain morphologic correlates of gender diversity among adolescents (assigned male or female at birth, separately) from a general population sample. Key findings are as follows. Global brain volumetric measures did not differ between adolescents with and without gender diversity. However, differences in specific brain regions depended on assigned sex at birth. In whole-brain, vertexwise analyses, we observed a thicker left inferior temporal gyrus cortex among adolescents assigned male with gender diversity compared with those assigned male who did not report gender diversity. Efforts to develop a quantitative scale for gender do not aim to develop a “diagnostic test” to confirm a person’s gender identity, but rather to elucidate individual differences in gender experiences.^8^ Similarly, our goal was not to uncover a mechanism that can be “fixed” to prevent gender diversity. Rather, we hope that by shedding light on the neurobiology of gender diversity among adolescents, progress can be made toward destigmatization, greater acceptance, and improved quality of life for individuals with diverse gender identities.^36^

The literature highlights substantial individual and societal burden for adolescents with gender diversity, possibly due to emotional distress resulting from living with gender dysphoria or from negative social interactions.^3^ Previous studies have shown that the development of brain structure and function is shaped by a complex interaction between prenatal and postnatal environmental factors continuously affecting the neural architecture throughout the lifetime.^37^ An earlier study in gender-diverse young adults found differences in subcortical brain volumes only.^16^ We similarly observed no global brain differences between adolescents with and without reports of gender diversity. We note that our study applied a population-based approach and examined gender diversity in adolescents. Such a population-based approach decreases the potential for selection bias, especially compared with studies with help-seeking populations.

Understanding of neurobiological underpinnings may inform the development of gender diversity and potential co-occurring behavioral and emotional problems. We observed that compared with youths who did not report gender diversity, adolescents assigned male at birth who reported gender diversity had a thicker inferior temporal gyrus cortex—an area that has been implicated in a wide array of functions, including language and semantic memory processing, visual perceptions, and multimodal sensory integration.^38^ Changes in cortical thickness may be attributable to a number of factors, such as synaptogenesis and dendritic arborization that can result in increased thickness, as well as synaptic pruning, cell death, and continued growth of white matter pathways into layer VI that can result in decreased cortical thickness.^39^ Multiple processes likely link gender diversity with brain morphology. In previous studies, the broad sense heritability of gender diversity in twin studies reached approximately 70% of the variance, with a similar magnitude of genetic and environmental factors between boys and girls,^9^ and did not vary with age.^9,40^ This broad sense heritability encompasses all genetic influences on gender diversity, including additive, dominance, epistasis, and gene-environment interaction effects. However, high heritability of a trait does not necessarily mean that the trait is not susceptible to environmental influences. Other potential mechanisms may include hormonal influences and other factors associated with the in utero environment, such as maternal-specific stressors that lead to direct physiologic changes or endocrine-mediated exposures with influences on fetal brain development.^10,11^ In our study, these mechanisms remain speculative, as a temporal direction was lacking and adolescents were scanned once and around the same age as reports of gender diversity. In addition to biological mechanisms, societal and cultural factors such as social relationships and cognitive learning can affect both gender diversity and brain development.^41,42^ Moreover, the substantial stress experienced by gender-diverse adolescents may affect their brain development.^43^

We adjusted all models for self-reported puberty because we anticipated that the brain correlates of gender diversity differ depending on pubertal status. Puberty is the period in which the male brain and the female brain increasingly diverge owing to the maturation of, among other brain regions, the prefrontal cortex underlying the development of behavioral control.^44,45^ Puberty is also associated with heightened neural sensitivity to emotional stimuli.^46^ Neural activity is driven by the onset of puberty, based on the assumption that pubertal hormones may increase sensitivity in these brain regions.^47^ However, incorporating puberty ratings did not change the associations observed between adolescent gender diversity and brain morphology.

### Limitations and Strengths

The following limitations of this cross-sectional study should be noted. First, this study was cross-sectional and lacked repeated measures of gender diversity and brain morphology. Second, our measure of gender diversity did not adequately cover the full spectrum, as we relied only on 2 items and did not directly measure gender identity or expression. In addition, we did not account for whether the participants socially transitioned. Third, parental genetic variation is associated with brain structural development; thus, biological vulnerabilities can be based on shared genetic characteristics, which may increase offspring susceptibility to the development of emotional and behavioral problems.^9^ Thus, accounting for parental genetic variation may enable us to determine the degree to which the association between gender diversity and brain structure is due to the contributions of genetic and environmental factors. Strengths of this study include its large population-based sample, broad spectrum of measured covariates, and use of 1 MRI machine to scan more than 2000 adolescents who also reported on gender diversity. Further, we included both maternal and paternal reports on child gender diversity and therefore investigated combined parent and offspring reports of gender diversity and brain structure associations.

## Conclusions

Our findings from this cross-sectional analysis of data from a population-based cohort suggest that within the general population, youths with gender diversity did not differ in relation to global brain measures from adolescents who did not endorse gender diversity. However, we observed that youths assigned male at birth—but not those assigned female at birth—had thicker cortices in the inferior temporal gyri compared with those who did not report gender diversity. Further research with repeated neuroimaging is required to measure the interplay between gender diversity and brain development from childhood into young adulthood and also to test the directionality of these associations.

## Notes

Supplement.
Data Sharing Statement
