# Supplementary material for: Gender Diversity and Brain Morphology Among Adolescents
Source: JAMA Netw Open. 2023 May 12;6(5):e2313139. doi: 10.1001/jamanetworkopen.2023.13139 (PMC10182431; doi:10.1001/jamanetworkopen.2023.13139)
Supplement: Supplement. — Data Sharing Statement [file jamanetwopen-e2313139-s001.pdf]

Data Sharing Statement

Xerxa. Gender Diversity and Brain Morphology Among Adolescents. *JAMA Netw Open*. Published May 12, 2023. doi:10.1001/jamanetworkopen.2023.13139

Data
Data available: No

Additional Information
Explanation for why data not available: Following The General Data Protection Regulation by the European Union, we will be able to share deidentified data under a data use agreement only.
